# Comparing different methods of indexing commercial health care prices

**DOI:** 10.1111/1475-6773.13242

**Published:** 2019-11-25

**Authors:** William C. Johnson, Kevin Kennedy

**Affiliations:** ^1^ Health Care Cost Institute Washington District of Columbia

**Keywords:** geographic/spatial factors/small area variations, Health Care Costs, health care financing/insurance/premiums

## Abstract

**Objective:**

To compare different methods of indexing health care service prices for the commercially insured population across geographic markets.

**Data Sources:**

Health Care Cost Institute commercial claims data from 2012 to 2016.

**Study Design:**

We compare price indices computed using methods with differing levels of computational intensity: weighted‐average versus regression‐based methods. We separately compute indices of the prices paid for set of common inpatient and set of common outpatient services in different markets across the United States using each type of method. We subsequently examined the variation of and correlations between the resulting index values.

**Data Collection/Extraction Methods:**

We computed health care service price indices separately using samples of inpatient and outpatient facility claims from 2012 to 2016 across 112 Core‐Based Statistical Areas. Within each category of services, claims were limited to members under the age of 65 with employer‐sponsored insurance. Both samples were limited to a common set of services that made up nearly 80 percent of the service use in the full sample every year.

**Principal Findings:**

We found that the methods studied produced highly correlated price indices (*r *> .94) with similar distributions across years for both inpatient and outpatient services.

**Conclusions:**

Our findings suggest that weighted‐average methods, which are much less computationally intensive, will generate results similar to regression‐based methods.

## 1 INTRODUCTION

Health care spending varies greatly throughout the United States. Unlike spending variation among the publicly insured, which is primarily attributable to variation in service use, there is evidence that commercial spending variation is also attributable to variation in service prices.^1^, ^2^, ^3^ These findings place a policy focus on understanding differences in health care prices across commercial health care markets. Different sources of price variation—such as provider or insurer market power, cost of living or other such factors—imply vastly different policy remedies. To better understand price variation and its potential causes, it is crucial to first determine how to measure prices in different geographic areas.

We assess how using different methods of benchmarking the transaction prices for health care services across geographic areas affects price measurement. Traditionally, researchers have used less computationally intensive average‐based measures that summarize the prices of a defined set of services.^4^ Related literature indexes prices by computing an average‐based measure of the prices for all services used to treat a given disease.^5^ These approaches, though, require the researcher to specify a set services which can be compared across areas and over time, as well as how to weight each service.

Alternatively, researchers can use more computationally intensive regression‐based approaches to measure medical prices across areas or providers.^1^ Regression‐based approaches have the advantages of not requiring the specification of a comparison set of services or service weights, more flexibly accounting for missing services, and better accounting for population heterogeneity between areas. These advantages, however, come at the cost of increased computational burden. When dealing with a dataset with hundreds of millions of claim observations for tens of thousands of distinct services, for example, the computational burden is not trivial.

We compare weighted‐average‐based methods with a regression‐based method of indexing commercial health care prices. We then empirically test whether these different methods produce substantially different price indices to inform the trade‐off of index performance and computational burden.

## 2 DATA

We leverage data from the Health Care Cost Institute (HCCI) on the allowed amounts paid for 250 million commercial claims spanning 112 Core‐Based Statistical Areas (CBSAs) across 43 states over 2012 to 2016. To construct a service‐level sample, we aggregate data from all claim lines associated with an individual on common dates for the same service. For inpatient admissions, we define a service by the Diagnostic Related Group (DRG) code. For outpatient services, we define the service by the combination of a Current Procedural Terminology (CPT) code and CPT code modifier. We define the total spending for a service as the sum of the allowed amounts for all associated claim lines. The allowed amount represents the actual amounts paid to a provider for a claim—including all insurer and individual out‐of‐pocket payments.

We limit our sample to individuals under the age of 65 with employer‐sponsored insurance. We exclude claims with extreme lengths of stay or costs. We use CBSAs as the geographic level to report our analysis. We also limit our analysis to CBSAs where the HCCI database has both sufficient data coverage and density of health care providers. For a complete description of our sample restrictions, see the Appendix S1.

In this study, we limit our analysis to inpatient admissions and outpatient facility services. For each category of services, we examine a subset of common services (see Appendix S1). Within both service categories, these common services account for more than 60 percent of all spending and almost 80 percent of service use in our sample in every year (see Appendix S1). Using each index method described below, we compute price index measures for each CBSA in our sample in each year for inpatient and outpatient services, separately. We define our base year as the first year in our sample (2012).

## 3 METHODS

### 3.1 Defining terminology

We calculate total spending on and use of each service. We define the average price for a “CBSA‐year” observation as the total spending on a service divided by the total number of times that service was performed in that CBSA in that year. We similarly calculate the average price of each service at the national‐year level as the sum of its total spending nationally divided by the total number of services performed nationally.

### 3.2 Weighted‐average approach I: Geometric average index

This approach computes a price index as the weighted geometric average of the ratio of the average price for each service in a CBSA‐year observation relative to the average price nationally in our base year:

$$\text{Geometric}\;\text{Average}\;{\text{Index}}_{\mathrm{tg}}=\prod_{s\in S}{\left[\frac{{\bar{\text{Price}}}_{\mathrm{tgs}}}{{\bar{\text{Price}}}_{\mathrm{Ts}}}\right]}^{\frac{\sum_{g\in G}{\text{Spend}}_{\mathrm{Tgs}}}{\sum_{s\in S}\sum_{g\in G}{\text{Spend}}_{\mathrm{Tgs}}}}$$

We weight each service *s* within our sample services *S* using the share of national total spending on our sample services accounted for by each service in our base year. Here, ${\bar{\text{Price}}}_{\mathrm{tgs}}/{\bar{\text{Price}}}_{\mathrm{Ts}}$ is the ratio of the average price of each service *s* in each CBSA *g* in each year *t*, and the average price of each service s nationally in our base year *T*. If a CBSA does not have any claims for a service, we impute the national average price as the average price in that CBSA; we impute a price ratio of one.

### 3.3 Weighted‐average approach II: Arithmetic average index

An alternative weighted‐average approach is an arithmetic average index.^4^ Using the same service weights as above, we compute a price index as the weighted arithmetic average of the ratio between the average price in each CBSA in each year and nationally in a base year (all terms are defined as before):

$${\text{Arithmetic Average Index}}_{\mathrm{tg}}=\sum_{s\in S}\left(\frac{{\bar{\text{Price}}}_{\mathrm{tgs}}}{{\bar{\text{Price}}}_{\mathrm{Ts}}}\right)*\left(\frac{\sum_{g\in G}{\text{Spend}}_{\mathrm{Tgs}}}{\sum_{s\in S}\sum_{g\in G}{\text{Spend}}_{\mathrm{Tgs}}}\right).$$

### 3.4 Regression‐based price index

Another type of approach to computing a price index is to estimate a regression‐based average price measure. Following previous literature, we compute such an estimate using the following specification for each claim *c* for service *s* provided in CBSA *g*, in year *t* using the following estimation equation^1^:

$${\text{Price}}_{\mathrm{csgt}}=\alpha +\beta X_{\mathrm{csgt}}+\gamma_{s}+\delta_{g}+\theta_{t}+\varepsilon_{\mathrm{csgt}}$$

Here, $X_{\mathrm{cgst}}$ is a vector of gender and age band indicator variables, $\gamma_{s}$ are service fixed effects, $\delta_{g}$ are CBSA fixed effects, $\theta_{t}$ are year fixed effects, and $\varepsilon_{\mathrm{csgt}}$ is an i.i.d. normally distributed error term (see Appendix S1).

Using these estimated coefficients, we predict the average price per service in each CBSA in each year holding constant the mix of patient demographic characteristics (age bands and gender) and service types. Here, $\bar{X}$ is a vector of sample means for each demographic indicator, and $\bar{s}$ is a vector of sample means for each service indicator:

$$\hat{{\text{Price}}_{\mathrm{gt}}}=\hat{\alpha }+\hat{\beta }\bar{X}+\bar{s}{\hat{\gamma }}_{s}+{\hat{\delta }}_{g}+{\hat{\theta }}_{t}$$

We also predict the average price per service nationally in each year holding constant the average mix of demographics, service types, and patients from each study CBSA. Here, all terms are defined as before and $\bar{g}$ is a vector of sample means for each CBSA indicator:

$${\hat{\text{Price}}}_{t}=\hat{\alpha }+\hat{\beta }\bar{X}+\bar{s}{\hat{\gamma }}_{s}+\bar{g}{\hat{\delta }}_{g}+{\hat{\theta }}_{t}$$

We then calculate a price index for each CBSA‐year observation as the ratio between the predicted price in each CBSA in each year and predicted price nationally in our base year:

$$\text{Regression}\;-\;\text{Based}\;{\text{Index}}_{\mathrm{gt}}=\frac{{\hat{\text{Price}}}_{\mathrm{gt}}}{{\hat{\text{Price}}}_{T}}$$

### 3.5 Comparison metrics

To compare the variation in the price indices we calculate using each method above, we examine the distributions of each index. We then compute pairwise Pearson correlation coefficients between the indices.

## 4 RESULTS

### 4.1 Price index variation

**Figure 1:**
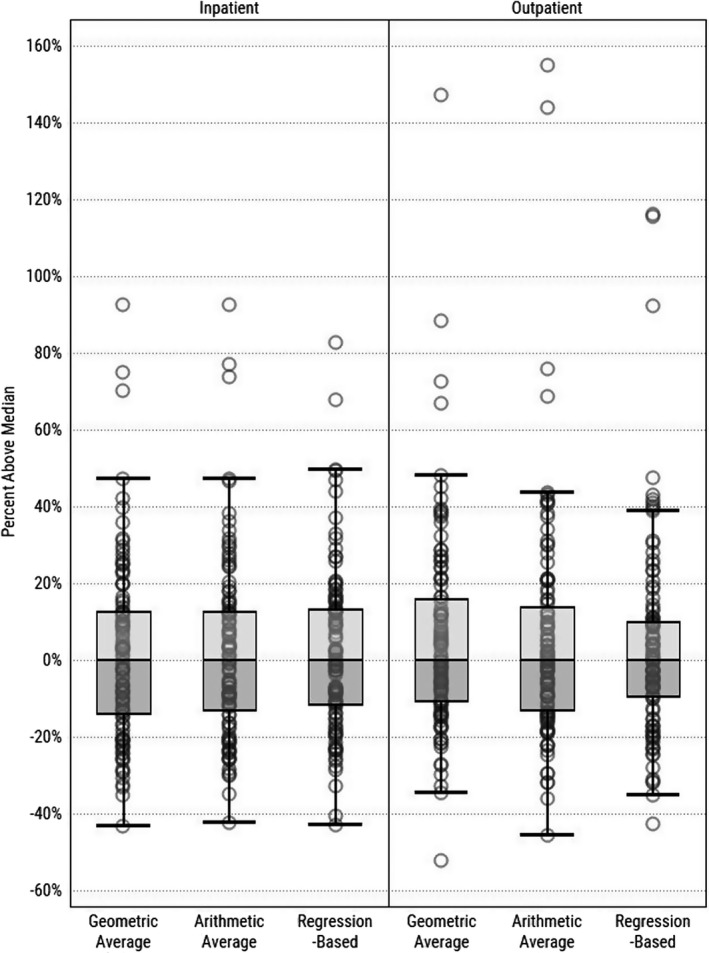
Comparing distributions of price indices calculated with different methods (2016)

Each approach produces wide variation in price index values across CBSAs. However, in our sample, the choice of approach had little effect on the shape of the resulting distribution of those values (Figure 1). For example, using the geometric average approach, in 2016, the CBSA with inpatient prices equal to the 75th percentile was 31 percent higher than the CBSA with inpatient prices equal to the 25th percentile. By comparison, the differences between the 75th and 25th percentile inpatient prices were 30 percent and 28 percent for the arithmetic average and regression‐based approaches, respectively.

### 4.2 Correlation of index measures using different methodologies

**Figure 2:**
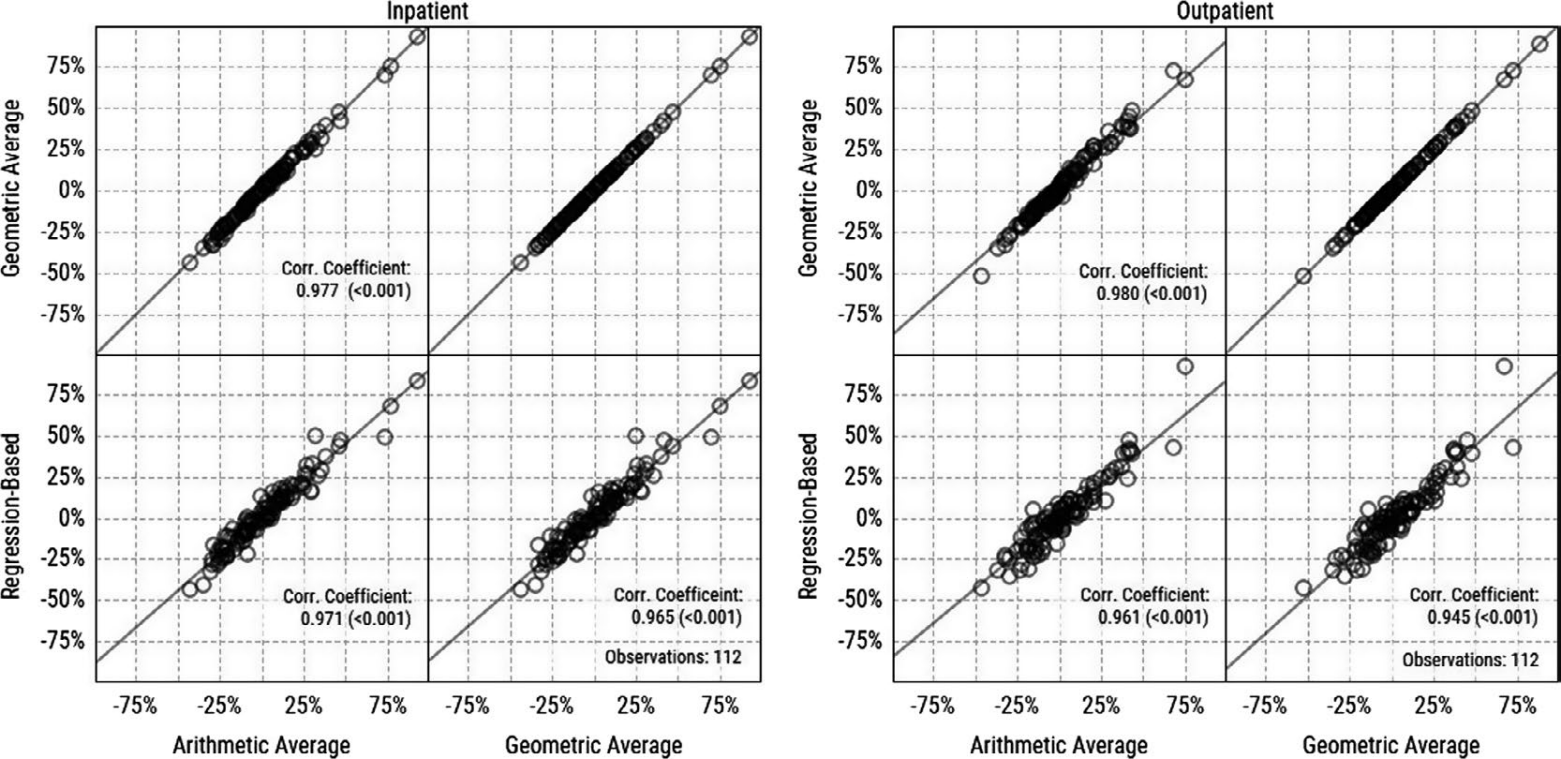
Pearson correlation coefficients among price indices calculated with different methods (2016). Note: Price indices reported as percent difference from national median

In addition to having similar distributions, we also find that the price indices calculated with different methods share a strong positive correlation. In other words, CBSAs with relatively high price levels using a particular approach have similarly high relative prices when using the other approaches. Pairwise scatter plots are presented in Figure 2 alongside corresponding Pearson's correlation coefficients for each set of index values across CBSAs in 2016 for each category of services. The pairwise correlations among the indices we compute are all above 0.96 for inpatient admissions and above 0.94 for outpatient services. These correlations are stable across all sample years (Table 1). As expected, the geometric and arithmetic average indices were the most closely related as they use the same service weights. Our results are robust to alternatively estimating our regression‐based price index using the natural logarithm of price as our dependent variable to allow for a skewed price distribution (see Appendix S1).

**Table 1:** Pairwise Pearson correlation coefficients between weighted‐average price indices and regression‐based price index over time

	2012	2013	2014	2015	2016
Inpatient services					
Arithmetic average	0.974 (<0.001)	0.976 (<0.001)	0.979 (<0.001)	0.976 (<0.001)	0.971 (<0.001)
Geometric average	0.975 (<0.001)	0.977 (<0.001)	0.975 (<0.001)	0.972 (<0.001)	0.965 (<0.001)
Outpatient services					
Arithmetic average	0.957 (<0.001)	0.965 (<0.001)	0.972 (<0.001)	0.972 (<0.001)	0.961 (<0.001)
Geometric average	0.931 (<0.001)	0.939 (<0.001)	0.945 (<0.001)	0.952 (<0.001)	0.945 (<0.001)

## 5 DISCUSSION

There are both advantages and disadvantages when computing price indices using each of the three methods discussed in this paper.

### 5.1 Weighted‐average approach I: Geometric average index

This approach has two primary advantages—it is computationally straightforward to implement and it allows for the price index to have convenient multiplicative properties. Per our definition of average price, this method makes it possible to decompose our price index as the ratio of similarly computed total spending and service use indices (see Appendix S1). This property facilitates comparing price and use levels both within and across CBSAs, decomposing spending variation into price and use variation, and comparing changes in price and use levels of the same set of services over time. Further, the multiplicative properties allow for intuitive interpretation of the ratios of any two index values. For example, the ratio of index values for CBSAs *A* and *B* in a given year is equal to the weighted geometric average of the ratio of average service prices between CBSAs *A* and *B* (see Appendix S1).

This method does contain some drawbacks. Namely, it requires selecting a “market basket” of services—defined by both the set of services compared and the weights assigned to each service. Indices computed with this method may be sensitive to both the set of services chosen and the weights imposed on each service.^4^ Using a market basket of services also requires observing the same set of services in each CBSA and in each year. In this context, missing data—where a given CBSA‐year combination has no observations for a given service(s)—requires adjusting the approach by either limiting the size of the market basket or imputing values. Our implementation of this method relies on imputing average prices in CBSAs that do not have any claims for a service in our market basket. This potentially biases the index value for CBSAs with imputed prices toward the national average.

An additional limitation when comparing the same market basket across areas and over time is that it does not flexibly allow for substitution—a common concern surrounding price indices. It is possible that individuals substitute away from high‐priced services. In this case, our market basket may overestimate price levels, price growth, or both. A related concern is that this approach compares the same services over time, so included services must be present in each period. If services that either enter or exit are disproportionately likely to have low prices, this will overestimate price growth over time.^6^

Another limitation of this approach is that it does not adjust for differences in populations across CBSAs. Heterogeneity in underlying population health could then be driving some of the heterogeneity in our price index values. For example, it is possible areas with sicker populations on average receive more expensive versions of the same service resulting in this index overstating such a CBSA's price level. This may be of particular concern when defining services using DRG codes, as we do with inpatient services.^7^

### 5.2 Weighted‐average approach II: Arithmetic average index

The main advantage of this approach, relative to the geometric average, is ease of interpretation. Here, the price index can be interpreted as the average price of a market basket where a certain number of each type of service (according to their weight) are included in the basket. Additionally, this approach is also not computationally intensive to implement.

The arithmetic average index shares the same limitations caused by selecting sample services and imposing service weights. In particular, it has the same potential limitation due to missing observations for sample services in some CBSA‐year combinations; necessitating either a narrower market basket or imputed data. One additional limitation relative to the geometric average index is that the arithmetic average index does not have the convenient multiplicative properties.

### 5.3 Regression‐based price index

The primary advantage of the regression‐based approach is that it more flexibly accounts for missing data. In this way, the regression‐based approach—unlike its weighted‐average counterparts—does not necessitate shrinking the size of the market basket or imputing data when there are no observations for services within the market basket in a given CBSA. Another advantage of the regression‐based approach is it can better account for population heterogeneity both across CBSAs and over time.

The main disadvantage of using a regression‐based method is it is computationally intensive. In the context of outpatient services, for example, there are tens of thousands of distinct outpatient services observed in our data. Estimating our regression specification would necessitate the inclusion of that many fixed effects to perform the same analysis on this full sample (rather than for the subset of common services which we studied). Further, when the samples include hundreds of millions of observations, the amount of time and computing power necessary to estimate such regressions can become prohibitive. As a result, researchers might be forced to either use a subset of the observations, limit to a subset of services, or both.

### 5.4 Assessing index methodology trade‐offs

The strong correlations between the regression‐based measure and both the weighted‐average indices potentially alleviate concerns about some of the shortcomings of the latter measures. This finding provides evidence adjusting for demographic factors potentially correlated with underlying population health (ie, age and gender) does not qualitatively change the price indices we compute.

Our findings also provide evidence that price indices for medical services are not sensitive to reasonable choices of market baskets—that is, both the set of services studied and the weights of services imposed. We observed strong correlations between indices which flexibly allow for substitution away from high‐priced services (regression‐based) and indices that do not (weighted‐average‐based), both across geographic areas and years. This supports the similar conclusions drawn by previous research.^4^

Each approach studied produces largely similar measures of relative commercial health care prices across geographies and over time. These findings echo previous work using a different data source and sample period, as well as surveying a new set of methods.^4^ In practice, we argue that these findings allow researchers to use less computational weighted‐average approaches knowing they will produce similar indices to regression‐based approaches. As big data become a mainstay of health services research, our findings provide empirical support for measuring commercial prices using methods with low computational intensity. Further, within weighted‐average approaches, the similarity in price indexes produced allows researchers to leverage the measure best suited for their purposes.

## References

[1] Cooper Z , Craig SV , Gaynor M , Van Reenen J . The price ain't right? Hospital prices and health spending on the privately insured. Q J Econ. 2019;134(1):51‐107. 32981974 10.1093/qje/qjy020 PMC7517591

[2] Newhouse JP , Kibria A , Mancher M , et al. Variation in Health Care Spending: Target Decision Making, Not Geography. Washington, DC: National Academies Press; 2013. 24851301

[3] Philipson TJ , Seabury SA , Lockwood LM , Goldman DP , Lakdawalla DN . Geographic variation in health care: the role of private markets. Brookings Pap Econ Act. 2010;2010(1):325‐355.

[4] Neprash HT , Wallace J , Chernew ME , McWilliams JM . Measuring prices in health care markets using commercial claims data. Health Serv Res. 2015;50(6):2037‐2047. 25772745 10.1111/1475-6773.12304 PMC4693848

[5] Dunn A , Liebman EB , Shapiro AH . Decomposing medical care expenditure growth In: Aizcorbe A, Baker C, Berndt E, Cutler D, eds. Measuring and Modeling Health Care Costs. Chicago, IL: University of Chicago Press; 2018:81‐111.

[6] Erickson T , Pakes A . An experimental component index for the CPI: from annual computer data to monthly data on other goods. Am Econ Rev. 2011;101(5):1707‐1738.

[7] Garthwaite C , Ody C . Beware false precision: the sources of health care spending growth are hard to identify. Health Aff. 2018 10.1377/hblog20181205.397704

