# Supplementary material for: Comparing different methods of indexing commercial health care prices
Source: Health Serv Res. 2019 Nov 25;55(1):113–8. doi: 10.1111/1475-6773.13242 (PMC6980960; doi:10.1111/1475-6773.13242)
Supplement: Supplementary file 2 [file HESR-55-113-s002.docx]

Online Appendix:

Measure Twice, Index Once? Comparing Different Methods for Indexing Commercial Health Care Prices

# *Sample Construction*

Using HCCI claims data, we constructed a sample of health care services provided in geographic areas across the country in each year from 2012 to 2016. The HCCI claims data are primarily organized at the claim line level. That is, for a service performed, the claim filed is broken up into multiple claim lines. To construct a service level sample from the claim line level data, we aggregated data from all claim lines associated with each service. This aggregated service will be referred to as a service claim.

## *Claim Line Inclusion Criteria*

### *Area Inclusion Criteria*

To be included in our sample, a claim line had to be associated with an individual from and a service provided within one of our analysis areas. We performed our analysis at the Core-Based Statistical Area (CBSA) level.

The CBSAs included in the study had to meet certain population, coverage, and utilization criteria. First, the sample CBSAs had to have a minimum average HCCI coverage of 10% over the 5-year period (2012-2016). Yearly HCCI coverage estimates were calculated by dividing HCCI’s member years (total member months divided by 12) within a CBSA by the American Community Survey (ACS) 5-year average employer sponsored insurance (ESI) population in that same CBSA.^[[1]](#footnote-1)^ Each sample CBSA had to have an average of at least 25,000 member years in the HCCI data from 2012-2016. Using data from the American Hospital Association (AHA), included CBSAs had to have a minimum of 5 distinct, non-governmental General Medical and Surgical Hospitals. Finally, we limited our sample to CBSAs which had a total of at least 125 admissions, taking place at 3 distinct hospitals within the HCCI data, for every year studied. This resulted in a final geographic sample of 112 CBSAs across 43 states.

*1.1.2 Member Inclusion Criteria*

We included all claim lines associated with members who were both in our study analysis CBSAs and a part of the sample population. For a member month to be included in the sample population, the member, in that given month, needed to be under the age of 65 and have an identifiable gender in the data. Additionally, they had to have ESI, non-individual coverage with one of the following plan types: Health Maintenance Organization, Preferred Provider Organization, Point of Service Plan, or Exclusive Provider Organization. Using these member months, we calculated our sample’s member year totals by CBSA and by year to determine the CBSA sample. We subsequently cleaned and analyzed their associated inpatient and outpatient, service claims. In total, our sample spans over 250 million service claims from 132 million member years across the study, 2012 - 2016.

## *1.2 Aggregating claim lines to service claims*

We define a service claim as all claim lines for an individual with common dates and service codes. We define service codes distinctly in each high-level service category (inpatient, outpatient). For inpatient claims, we define a service code as DRG codes. For outpatient claims, we define service codes as the combination of CPT code and CPT code modifier. For the remainder of this document, we use CPT code to refer to the combination of CPT code and CPT code modifier.

When aggregating claim lines to the service claim level, we summed all allowed amounts (the actual amount paid to for the claim) from each claim line associated with a particular service claim. Allowed amounts comprise both the insurer’s payment to a provider as well as any out-of-pocket spending (copayments, coinsurance, or deductibles) by the patient. We define the sum of the these allowed amounts as the total spending on a service claim.

##

## *1.3 Claim Inclusion Criteria*

We apply distinct inclusion exclusion criteria for claim lines from each service category (inpatient, outpatient). For inpatient services, we exclude inpatient service claims with overlapping lengths of stays. For example, if the same individual had two claims on the same date (e.g., one for a service code indicating Simple Pneumonia and Pleurisy (DRG 193) and another service code indicating Heart Failure and Shock (DRG 291) both service claims would be excluded from our sample). We also excluded inpatient service claims where any of the claim lines took place at a non-General Acute Care (GAC) hospital or if they were associated with a pre-Major Diagnostic Category (MDC) code. Included service claims needed to have consistent types of bill codes that indicated an inpatient hospital visit. Service claims were excluded if they did not take place at a GAC, non-governmental, non-military hospital found in the AHA data, or if there were claim lines which indicated the service claim took place at multiple hospitals.

For outpatient services, the sample was limited to service claims that consisted of claim lines with only the following type of bill codes: hospital outpatient, hospital laboratory services, ambulatory surgery center, any of the eight types of clinics (rural health, hospital based or independent renal dialysis center, freestanding, outpatient rehabilitation, comprehensive outpatient rehabilitation, community mental health, federally qualified health, and other), or a freestanding emergency medical facility. We found the number of units most commonly associated with each CPT code in each year. Service claims with unit counts differing from their corresponding CPT code and year combination’s mode of units were excluded to ensure reported prices were the price of the most typical visit for that service code in that year.

Additionally, we excluded claims with extreme length of stay or costs. We only included inpatient service claims with lengths of stay under 180 days. Outpatient services had to occur on a single day. Across both categories, we excluded service claims with a total charge amount less than or equal $1 or a total spending amount (the actual amount paid to the providers including any patient cost sharing) less than or equal to $1. We also excluded services with a total spending to total charge ratio less than or equal to 20 percent. Finally, the inpatient sample was further trimmed by removing the top and bottom 1% of service claims based off their total spending.

*1.4 Identifying a Set of Common Services*

We constructed a set of common services for each service category. To do so, we first aggregated the number of service claims for each service code within each calendar year for each category. We restricted the service codes included in our set of common service codes to meet two criteria: (1) a service code must appear in each year of our data and (2) a service code must be present in at least 80% of CBSAs in our sample. For each category, we then constructed a set of the most common service codes (“common services”) observed in our base year (2012) meeting our inclusion criteria:

- Inpatient Services: the 100 DRG codes with the highest share of nationwide inpatient admissions in 2012
- Outpatient Services: the 500 CPT codes with the highest share of nationwide outpatient procedures in 2012

Appendix Table 1: Share of Total Spending, Services Accounted for by Sample Services

|  |  |  |  |  |  |
| --- | --- | --- | --- | --- | --- |
|  | Inpatient | |  | Outpatient | |
|  |  |  |  |  |  |
|  | Percent of Spending | Percent of Services |  | Percent of Spending | Percent of Services |
| Year |  |  |  |  |  |
| 2012 | 63.0% | 78.1% |  | 61.0% | 80.1% |
| 2013 | 63.2% | 78.6% |  | 60.6% | 79.8% |
| 2014 | 62.6% | 79.0% |  | 60.3% | 78.8% |
| 2015 | 62.1% | 79.0% |  | 60.1% | 77.2% |
| 2016 | 62.2% | 79.0% |  | 59.0% | 76.4% |
|  |  |  |  |  |  |

*2. Properties of Geometric Average Indices*

*2.1 Comparing Spending, Price, Use Indices*

For a given category of services, when using the geometric average approach we compute our price index for CBSA *g* in year *t* as the weighted geometric average of all services *s* in our set of common services *S*:

$$P_{tg}= \prod_{s \in S} \left[ \frac{\bar{Price}_{tgs}}{\bar{Price}_{Ts}} \right]^{w_{s}}$$

Here for simplicity $w_{s}$ represents the weight for each service *s* defined by its expenditure share in our base year:

$$w_{s}=\frac{\sum_{g\in G} {Spend}_{Tgs}}{\sum_{s\in S} \sum_{g\in G} {Spend}_{Tgs}}$$

Recall that we defined “average price” as the ratio of the total spending on a service in a given geographic area and year divided by the number of times that service was performed in that geographic area and year:

$$\bar{Price}_{tgs}=\frac{{Spend}_{tgs}}{{Use}_{tgs}}=\frac{\left( \frac{{Spend}_{tgs}}{{Avg. Member Years}_{tg}} \right)}{\left( \frac{{Use}_{tgs}}{{Avg. Member Years}_{tg}} \right)}=\frac{PC {Spend}_{tgs}}{PC {Use}_{tgs}}$$

Average price is also equal to the ratio of per capita spending and use on service *s* in CBSA *g* in year *t*. For example as shown above, we could adjust total spending and service use to compute measures of per capita spending and use by dividing by the average number of member years in the relevant CBSA and year. The average member year numbers could be computed using the methodology described above (Appendix – Section 1.1.2).

Thus, given our sample of services performed in our sample CBSAs in our sample years, we could compute geometric average per capita spending ($T_{tg})$ and per capita use ($U_{tg})$ indices to match our geometric average price index:

$$T_{tg}= \prod_{s \in S} \left[ \frac{{PC Spend}_{tgs}}{PC {Spend}_{Ts}} \right]^{w_{s}}$$

$$U_{tg}= \prod_{s \in S} \left[ \frac{{PC Use}_{tgs}}{PC {Use}_{Ts}} \right]^{w_{s}}$$

We could then decompose our price index as the ratio of the per capita spending and use indices:

$$P_{tg} = \prod_{s \in S} \left[ \frac{\bar{Price}_{tgs}}{\bar{Price}_{Ts}} \right]^{w_{s}}= \prod_{s \in S} \left[ \frac{\left( \frac{{PC Spend}_{tgs}}{PC {Spend}_{Ts}} \right)}{\left( \frac{{PC Use}_{tgs}}{PC {Use}_{Ts}} \right)} \right]^{w_{s}}= \frac{\prod_{s \in S} \left[ \frac{{PC Spend}_{tgs}}{PC {Spend}_{2012s}} \right]^{w_{s}}}{\prod_{s \in S} \left[ \frac{{PC Use}_{tgs}}{PC {Use}_{2012s}} \right]^{w_{s}}}= \frac{T_{tg}}{U_{tg}}$$

*2.2 Comparing Two Index Values*

Due to the multiplicative properties of a geometric average index, the ratio of any two index values (i.e., two CBSAs or one CBSA in two different years) is equal to the geometrically weighted average of the ratio of those two observations prices (ratio of two CBSAs’ prices, or ratio of the CBSA’s prices in each years) for each service.

For example, consider comparing the prices of CBSA *A* and CBSA *B*. Let $P_{tA}$ be the price index computed for CBSA *A* and$P_{tB}$ be the price index computed for CBSA *B*:

$$\frac{P_{tA}}{P_{tB}}= \frac{\prod_{s \in S_{F}} \left[ \frac{\bar{Price}_{tAs}}{\bar{Price}_{Ts}} \right]^{w_{s}}}{\prod_{s \in S_{F}} \left[ \frac{\bar{Price}_{tBs}}{\bar{Price}_{Ts}} \right]^{w_{s}}} =\prod_{s \in S_{F}} \left[ \frac{\bar{Price}_{tAs}}{\bar{Price}_{tBs}} \right]^{w_{s}}$$

Alternatively, consider comparing the prices of CBSA *A* in year *0* and year *1*. Let $P_{0A}$ be the price index computed for CBSA *A* in year 0 and$P_{1A}$ be the price index computed for CBSA *A* in year *1*:

$$\frac{P_{1A}}{P_{0A}}= \frac{\prod_{s \in S_{F}} \left[ \frac{\bar{Price}_{1As}}{\bar{Price}_{Ts}} \right]^{w_{s}}}{\prod_{s \in S_{F}} \left[ \frac{\bar{Price}_{0As}}{\bar{Price}_{Ts}} \right]^{w_{s}}} =\prod_{s \in S_{F}} \left[ \frac{\bar{Price}_{1As}}{\bar{Price}_{0As}} \right]^{w_{s}}$$

*3. Properties of Regression-Based Price Measure*

*3.1 Computing our Regression-Based Price Measure*

Following Cooper et al. (2018), we compute our regression-based price measure by first calculating an estimate for the price per service using the following specification for each claim *c* for service *s* provided in CBSA *g*, in year *t* using the following estimation equation:

$${Price}_{csgt}=\alpha+ \beta X_{csgt}+\gamma_{s}+\delta_{g}+ \theta_{t}+\varepsilon_{csgt}$$

Here $X_{cgst}$ is a vector of gender and age band indicator variables: gender (male, female), age bands (0-18, 19-24, 25-34, 35-44, 45-54, 55-64). $\gamma_{s}$ are service fixed effects, $\delta_{g}$ are CBSA fixed effects, $\theta_{t}$ are year fixed effects, and $\varepsilon_{csgt}$ is an i.i.d. normally distributed error term.

Using these estimated coefficients, we predict the average price per service in each CBSA in each year holding constant the mix of patient demographic characteristics (age bands and gender) and service types. Here $\overline{X}$ is a vector of sample means for each demographic indicator, $\overline{s}$ is a vector of sample means for each service indicator:

$$\hat{Price}_{gt}=\hat{\alpha} + \hat{\beta}\overline{X}+{\overline{s}\hat{\gamma}}_{s}+\hat{\delta}_{g}+ \hat{\theta}_{t}$$

We also predict the average price per service nationally in each year holding constant the average mix of demographics, service types, and patients from each study CBSA. Here, all terms are defined as before and $\bar{g}$ is a vector of sample means for each CBSA indicator:

$$\hat{Price}_{t}=\hat{\alpha} + \hat{\beta}\overline{X}+{\overline{s}\hat{\gamma}}_{s}+{\bar{g}\hat{\delta}}_{g}+ \hat{\theta}_{t}$$

We then calculate a price index for each CBSA-year observation as the ratio between the predicted price in each CBSA in each year and predicted price nationally in our base year:

${Regression Based Index}_{gt}= \frac{\hat{Price}_{gt}}{\hat{Price}_{T}}$

*3.2 Robustness Check of Regression-Based Price Measure using the Natural Logarithm of Price*

To ensure that outliers were not resulting in potentially skewed price distributions, we also estimated the regression-based approach with the natural logarithm of price as the dependent variable. As seen in Appendix Table 2 below, there was very little difference in our results when substituting log(price) in for price as the outcome variable, especially for inpatient services.

Appendix Table 2: Comparison of Price Indices Computed by Different Methods, 2016


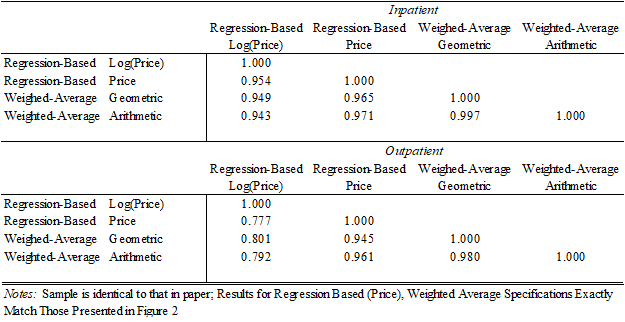


While there is slightly more of a difference for outpatient services, the resulting price indices were still very highly correlated across methods. For both inpatient and outpatient services, it appears that the minimal differences due to the skewed distribution would not affect our conclusions.

1. Two different 5-year estimates were provided by the American Community Survey (ACS). For 2012-2014, we used the 2010-2014 5-year ACS estimate as the denominator when determining HCCI percent coverage in that CBSA in each of those three years. For 2015 and 2016, we used the 2012-2016 5-year ACS estimate as the denominator. [↑](#footnote-ref-1)
